# Molecular hydrogen suppresses glioblastoma growth via inducing the glioma stem-like cell differentiation

**DOI:** 10.1186/s13287-019-1241-x

**Published:** 2019-05-21

**Authors:** Meng-yu Liu, Fei Xie, Yan Zhang, Ting-ting Wang, Sheng-nan Ma, Peng-xiang Zhao, Xin Zhang, Tyler W. Lebaron, Xin-long Yan, Xue-mei Ma

**Affiliations:** 1 College of Life Science and Bio-engineering, Beijing University of Technology, Beijing, 100124 China; 2 Beijing Molecular Hydrogen Research Center, Beijing, 100124 China; 3 Affiliated Bayi Brain Hospital, The Seventh Medical Center of PLA General Hospital, Beijing, 100700 China; 4 Correction is Molecular Hydrogen Institute, Enoch, UT USA; 5 Center of Experimental Medicine, Institute for Heart Research, Slovak Academy of Sciences, Bratislava, Slovak Republic

**Keywords:** Molecular hydrogen, Glioblastoma, Glioma stem-like cell, Cancer cell stemness

## Abstract

**Background:**

Glioblastoma (GBM) is the most common type of primary malignant brain tumor. Molecular hydrogen has been considered a preventive and therapeutic medical gas in many diseases including cancer. In our study, we sought to assess the potential role of molecular hydrogen on GBM.

**Methods:**

The in vivo studies were performed using a rat orthotopic glioma model and a mouse subcutaneous xenograft model. Animals inhaled hydrogen gas (67%) 1 h two times per day. MR imaging studies were performed to determine the tumor volume. Immunohistochemistry (IHC), immunofluorescence staining, and flow cytometry analysis were conducted to determine the expression of surface markers. Sphere formation assay was performed to assess the cancer stem cell self-renewal capacity. Assays for cell migration, invasion, and colony formation were conducted.

**Results:**

The in vivo study showed that hydrogen inhalation could effectively suppress GBM tumor growth and prolong the survival of mice with GBM. IHC and immunofluorescence staining demonstrated that hydrogen treatment markedly downregulated the expression of markers involved in stemness (CD133, Nestin), proliferation (ki67), and angiogenesis (CD34) and also upregulated GFAP expression, a marker of differentiation. Similar results were obtained in the in vitro studies. The sphere-forming ability of glioma cells was also suppressed by hydrogen treatment. Moreover, hydrogen treatment also suppressed the migration, invasion, and colony-forming ability of glioma cells.

**Conclusions:**

Together, these results indicated that molecular hydrogen may serve as a potential anti-tumor agent in the treatment of GBM.

## Background

Glioblastoma (GBM) is the most common type of primary malignant brain tumor and is characterized by rapid proliferation, diffusive invasion into normal brain tissues, and strong chemoresistance [1]. The current standard therapy for GBM is maximal resection followed by radiotherapy with concomitant and adjuvant temozolomide [2]. The invasive properties of GBM are a major obstacle for curative treatment, since it makes complete surgical resection impossible and causes the tumor recurrence after therapy [3]. Despite years of research investigating potentially new therapies for GBM, current therapeutic strategies are insufficient to control the disease, as is reflected by 1-year relative survival of 37.4% and 5-year survival of 4.9% [4]. Therefore, the development of new methods for GBM treatment is particularly important.

Molecular hydrogen has been considered as a preventive and therapeutic medical gas since Ohsawa et al. reported that inhalation of 1–4% hydrogen gas markedly reduced the sizes of cerebral infarction in rats [5]. The potential benefits of H_2_ have now been demonstrated in over 170 different human and animal disease models [6]. Recently, the effects of molecular hydrogen in cancer have attracted significant attention. Dole et al. first reported the therapeutic role of hydrogen on cutaneous squamous cell carcinoma in *Science* in 1975. They found that hyperbaric treatment of 97.5% hydrogen gas at a total pressure of 8 atm for 2 weeks could markedly induce tumor regression in mice [7]. Due to the special conditions of hyperbaric hydrogen treatment, subsequent research on the anti-cancer effect of hydrogen has been stagnant until the biological effects of low concentration hydrogen were reported on by Ohsawa et al. in 2007. Several years later, Saitoh et al. reported that neutral pH hydrogen-enriched electrolyzed water (NHE water) could achieve inhibition of tumor growth and of tumor invasion in an in vitro cell model [8]. Subsequent in vitro studies showed that molecular hydrogen could inhibit cancer cell proliferation [9–11], migration, invasion [9, 11], and colony formation [11] and induce cancer cell apoptosis [9, 10]. Several in vivo studies have demonstrated that molecular hydrogen could prevent carcinogenesis [12–14], inhibit cancer progression [9, 11], relieve the side effects of chemotherapy or radiotherapy [15–17], and enhance the anti-tumor effects of chemotherapeutic drugs [10, 18]. Although molecular hydrogen has shown potential in the field of cancer therapy, its anti-cancer properties are limited to only a few tumor types, and the underlying molecular mechanisms remain to be established.

In this study, we investigated the possible therapeutic effects of molecular hydrogen on GBM. Both in vivo and in vitro experimental models were used to evaluate the potential role of molecular hydrogen. The mechanisms underlying the effects of hydrogen have also been investigated.

## Materials and methods

### Animals and tumor cell lines

Male Wistar rats (8 weeks old, 170–180 g) and female BALB/c nude mice (8 weeks old, 20–24 g) were purchased from Vital River Laboratory Animal Technology Co., Ltd. (Beijing, China). Animals were maintained under standard conditions at 22 °C to 25 °C with a 12-h light-dark cycle and were fed a normal diet. All procedures were conducted in accordance with the Regulations for the Administration of Affairs Concerning Experimental Animals (China).

Rat C6 glioma cells and human U87 cells were obtained from the American Type Cell Collection (ATCC, Manassas, VA, USA). Cells were routinely cultured at 5% CO_2_ and 37 °C in DMEM/F12 medium (Gibco, NY, USA) supplemented with 10% fetal calf serum (Gibco, NY, USA) and 1% penicillin and streptomycin (Gibco, NY, USA).

### Rat C6 glioma model

Rats were fasted for 1 day before the experiments. They were then anesthetized by intraperitoneal injection of 10% chloral hydrate (3 mL/kg; Sigma, USA) and fixed in a stereotactic apparatus. The skin of the scalp was then incised in the midline of the skull with surgical scissors. Then, a hole was made in the cranial bone 1 mm posterior to the bregma and 3 mm lateral to the sagittal suture. A 30-gauge needle with a microsyringe was inserted to a depth of 7 mm from the skull surface; C6 glioma cells (1 × 10^6^) in 10 μL phosphate-buffered saline (PBS) were injected stereotactically. The injection was conducted over 10 min, after which the needle was held in position for 5 min and then gradually withdrawn over 3 min to prevent the backward flow of the solutions. After implantation of glioma cells, the rats were randomly divided into two groups including the hydrogen inhalation group (HI) and control group (CTRL).

### Mouse U87 subcutaneous model

BALB/c nude mice were injected subcutaneously with 1 × 10^6^ viable U87 cells. After injection, the mice were randomly assigned to two groups including the hydrogen inhalation group (HI) and control group (CTRL). Tumor volumes were measured on a weekly basis using the following formula: volume = width^2^ × length × 0.4.

### Inhalation of hydrogen gas

A transparent closed box (20 cm × 18 cm × 15 cm) was connected to an AMS-H-3 hydrogen-oxygen nebulizer machine (Asclepius Meditec Inc., Shanghai, China), which produces 67% H_2_ and 33% O_2_ (*V*/*V*). Hydrogen treatment was given on the second day of the rat C6 glioma or mouse U87 subcutaneous model establishment until the end of the experiment. Animals were placed in this box and inhaled the mixed air for 1 h two times per day. During this inhalation, mice were awake and freely moving. Thermal trace GC ultra-gas chromatography (Thermo Fisher, MA, USA) was used to monitor the concentration of hydrogen gas in the closed box.

### Hydrogen-rich medium treatment

A hydrogen-rich medium was produced by placing a metallic magnesium stick (Doctor SUISOSUI®; Friendear Inc., Tokyo, Japan) into DMEM/F12 medium (final hydrogen concentration 0.55–0.65 Mm). The hydrogen concentration was monitored by using a needle-type Hydrogen Sensor (Unisense A/S, Aarhus, Denmark).

### MR imaging

MR imaging studies were performed with the Bruker 7.0 T ClinScan high-field small-animal MRI system (Bruker BioSpin, Ettlingen, Germany). The tumor-bearing rats were anesthetized with 2% isoflurane in 2 L min^−1^ of oxygen and maintained at a normal body temperature. The T2-weighted MR images were acquired for each rat on days 17 and 26 after tumor transplantation using a conventional spin-echo sequence with the following parameters: TR = 3140 ms, TE = 37 ms, bandwidth = 130 Hz, flip angle = 180°, FOV = 4 cm × 4 cm, and slice thickness = 1 mm.

### Histology and immunohistochemistry

Glioma tissues were embedded in paraffin and then cut into 8-μm-thick sections. Sections were treated with 3% hydrogen peroxide for 10 min to inactivate endogenous peroxidases, followed by incubation with 10% normal goat serum. After the blocking serum was removed, sections were incubated overnight at 4 °C with primary antibodies including anti-CD34 (1:200; Abcam), anti-Ki67 (1:200; Abcam), anti-CD133 (1:50; Biobyt), and anti-Nestin (1:100; Millipore). Detection was performed using an HRP-conjugated secondary antibody followed by colorimetric detection using a DAB kit. All data were evaluated by blinded investigators.

### Immunofluorescence

Cells were seeded on coverslips, fixed with 4% paraformaldehyde (Sigma-Aldrich) for 10 min, permeabilized with 0.1% Triton X-100 in PBS, and blocked with 1% BSA for 1 h. Subsequently, cells were incubated overnight at 4 °C with primary antibodies including anti-GFAP (1:200; Abcam) and anti-CD133 (1:50; Biobyt). Cells were then incubated with FITC-conjugated secondary antibody (Abcam) for 1 h. The nuclei were stained with Hoechst, and the fluorescence images obtained with an Olympus IX71 inverted microscope.

### Flow cytometry

The expression of CD133 in cultured cells was analyzed by flow cytometry. In brief, cells were incubated for 10 min with 10% horse serum in PBS at 4 °C, followed by incubation with primary anti-CD133 (1:200; Biobyt) at 4 °C for 30 min. After centrifugation, the collected cells were incubated with FITC-conjugated secondary antibody (Abcam) for 20 min. After staining, cells were subjected to flow cytometry for analysis using a flow cytometer (Guava easyCyte 8HT; Millipore).

### Sphere formation assay

Cells were collected and washed to remove serum, then suspended in serum-free DMEM or MEM/EBSS medium at a density of 2.0 × 10^3^/mL. Five hundred cells per well were counted and seeded onto ultra-low attachment six-well plates (Corning, Corning, NY) for the sphere formation assay. Cells were cultured in normal serum-free medium or hydrogen-rich serum-free medium with B27 supplement (GIBCO, Grand Island, NY), 20 ng/mL of epidermal growth factor (EGF) (Pepro Tech Inc., Rocky Hill, NJ), and 20 ng/mL of basic fibroblast growth factor (bFGF) (Pepro Tech). The medium was changed every 3 days. Cells were incubated for 10 days, and spheres with a diameter > 50 μm were counted.

### Cell migration assay

The cell migration ability was examined using a wound-healing assay. Briefly, cells were seeded in six-well plates at a concentration of 1.0 × 10^6^/well and cultured for 24 h. A plastic pipette tip was used to scratch a line across the cell surface in each plate. The remaining cells were washed three times with PBS to remove the floating cells and debris, followed by incubation for 48 h in normal complete medium or hydrogen-rich medium. The images of the healing process were photographed digitally at the time point of immediately following and 24 h after wounding. The wound-healing assay was performed in three independent experiments.

### Cell invasion assay

The cell invasion ability was determined using BD matrigel invasion chambers according to the manufacturer’s protocol. Briefly, the top chambers with polycarbonate filters (8-μm pore size; Costar, Acton, MA) were coated with 50 μL of Matrigel (0.8 μg/μL, 37 °C, 2 h; BD Biosciences, San Diego, CA). 1 × 10^5^ cells in a 100-μL serum-free medium were seeded to the top chamber, and a 650-μL normal complete medium with or without hydrogen was added to the bottom. The cells were allowed to migrate through the porous membrane at 37 °C for 48 h. Then, cells in the upper surface of the chamber were completely removed by cotton swabs. The cells on the lower surface were stained with 0.1% (*w*/*v*) crystal violet after fixation, and five random fields from each insert were counted at × 100 magnifications. The invasion assay was conducted in triplicate-independent experiments.

### Colony formation assay

One thousand cells per well were counted and seeded in six-well plates. The plates were incubated for 14 days in a normal complete medium either with or without hydrogen, and then the cells were fixed by 4% paraformaldehyde and stained using 0.1% crystal violet. Colonies were counted only if they included at least 15 cells. Triplicate-independent experiments were performed, and all the visible colonies were calculated manually.

### Statistical analysis

Groups from cell culture and in vivo experiments were compared using two-tailed Student’s *t* tests, and results are presented as means ± SEM. All statistical analyses were performed using GraphPad Prism 6.01. A value of *p* < 0.05 was considered significant.

## Results

### Hydrogen inhalation inhibited glioma growth in vivo

**Fig. 1:**
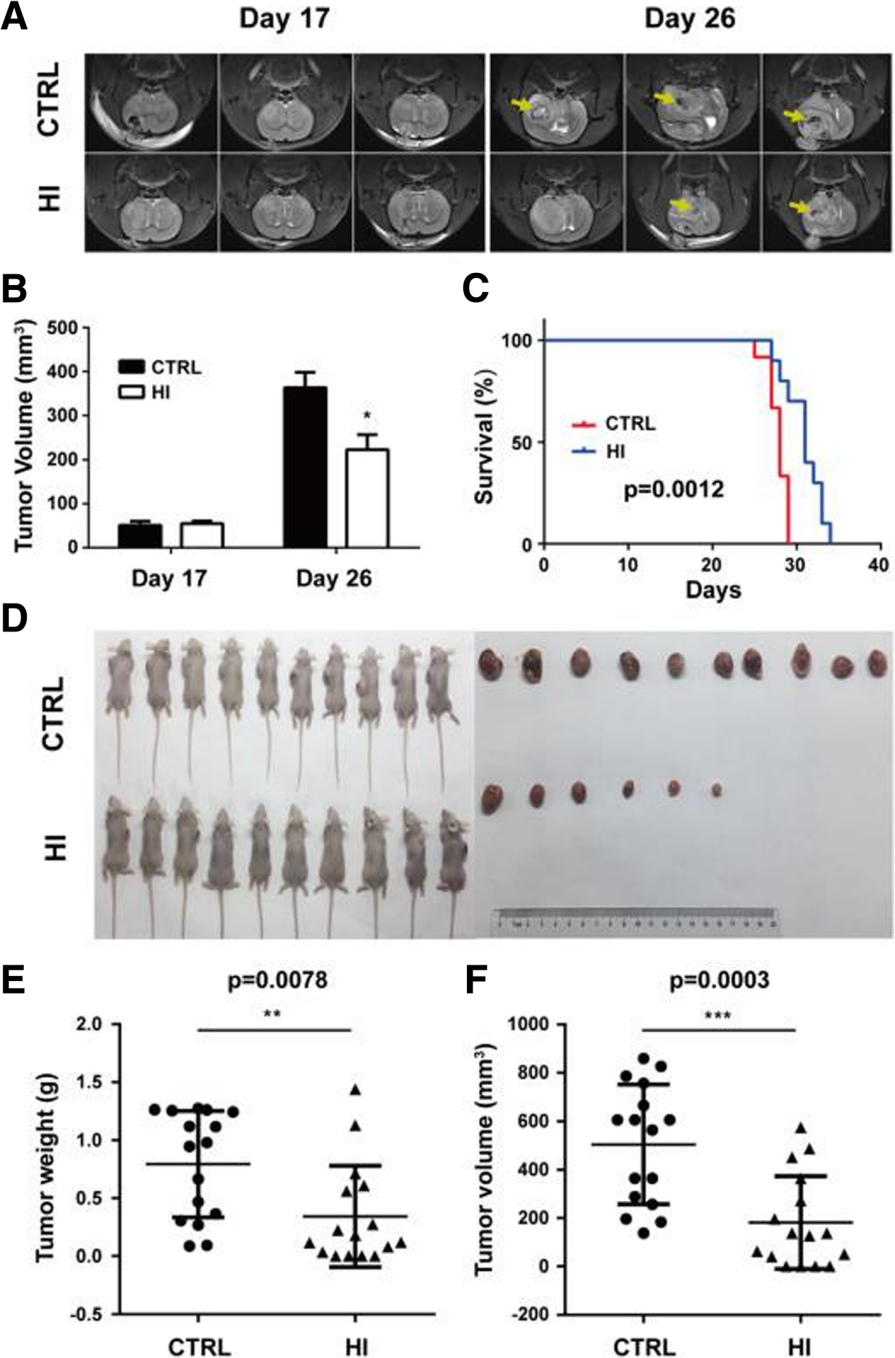
Hydrogen inhalation inhibited glioma cell growth in vivo. MR imaging was performed to evaluate the tumor growth of glioma in a rat C6 glioma model. The representative images were captured (**a**) and quantified (**b**). Hydrogen inhalation induced prolongation of median survival (**c**). Hydrogen inhalation also inhibited the tumor growth in a U87 subcutaneous mouse model (**d**). Both the tumor weight (**e**) and tumor volume (**f**) were decreased after hydrogen inhalation. *p < 0.05, **p < 0.01, ***p < 0.001 compared to the CTRL group. CTRL, control; HI, hydrogen inhalation

The effect of molecular hydrogen on tumor growth of glioma cells was first evaluated in a rat C6 glioma model. MR imaging results show no significant difference in tumor size between the HI group (54.76 ± 6.07) and the control group (51.23 ± 9.11) on day 17 after C6 cell implantation. However, on day 26, the tumor volume was significantly decreased in the HI group compared to the control group (223.3 ± 33.83 mm^3^ vs. 363.3 ± 34.80 mm^3^, *p* = 0.045) (Fig. 1a, b). In addition, hydrogen inhalation also induced prolongation of median survival (28.00 days vs. 31.00 days, *p* = 0.0012) (Fig. 1c).

To confirm the effect of molecular hydrogen on glioma growth, a mouse U87 subcutaneous model was used. As shown in Fig. 1d–f, hydrogen inhalation significantly reduced tumor weight (0.34 ± 0.11 g vs. 0.79 ± 0.11 g, *p* = 0.0078) and volume (181.30 ± 48.02 mm^3^ vs. 504.20 ± 61.71 mm^3^, *p* = 0.0003) as compared to controls.

### Hydrogen treatment suppressed the stemness of glioma cells

**Fig. 2:**
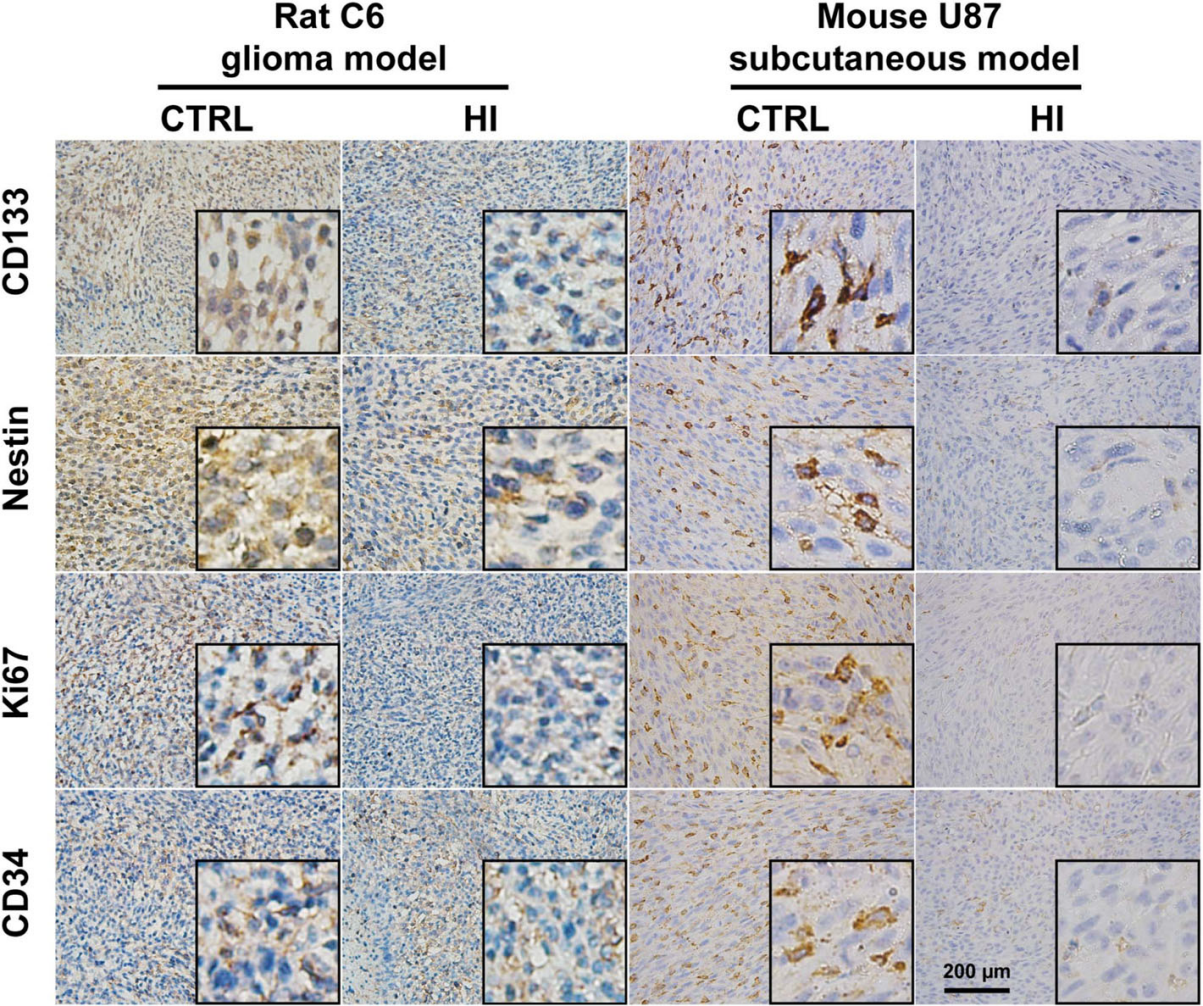
The expression of biomarkers were changed after hydrogen inhalation. Hydrogen inhalation decreased the expression of CD133, Nestin, Ki67, and CD34 in both rat C6 glioma and mouse U87 subcutaneous models. CTRL, control; HRM, hydrogen-rich medium

To identify the potential effects of molecular hydrogen on the stemness of glioma cells, the expression of stemness markers were examined both in rat C6 glioma and mouse U87 subcutaneous models. Immunohistochemistry (IHC) staining showed that the expression of CD133 and Nestin was significantly decreased in the HI group as compared to control either in a rat C6 or a mouse U87 model (Fig. 2). The expression of Ki67 and CD34 was also markedly decreased in the HI group compared to the control (Fig. 2).

**Fig. 3:**
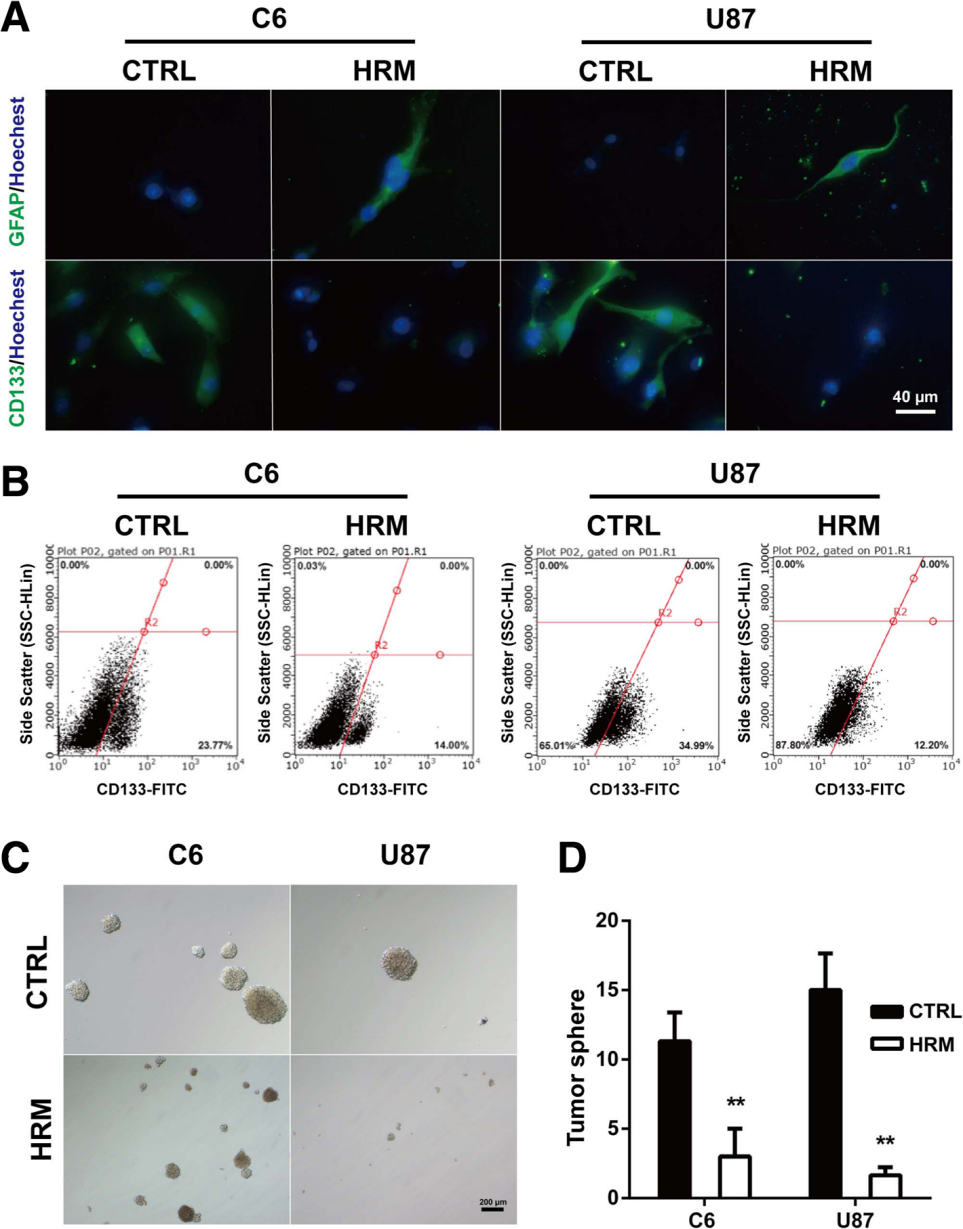
Hydrogen treatment attenuated the stemness of glioma cells. The expression levels of GFAP and CD133 in glioma cells were assessed with immunofluorescence staining after hydrogen treatment (**a**)**.** The CD133-positive cell number was determined using flow cytometry analysis after hydrogen treatment (**b**). The cancer stem cell self-renewal capacity of glioma cells was determined by using sphere formation assays. The representative images were captured (**c**) and quantified (**d**). **p < 0.01 compared with CTRL group. GFAP, glial fibrillary acidic protein; CTRL, control; HRM, hydrogen-rich medium

To confirm the effect of molecular hydrogen on the stemness of glioma cells, immunofluorescence was performed in C6 and U87 cells in vitro. Hydrogen-rich medium induced a marked reduction of CD133 expression both in C6 and U87 cells (C6, 26.08 ± 1.93 vs. 45.02 ± 3.47, *p* = 0.0088; U87, 26.11 ± 3.89 vs. 51.81 ± 3.09, *p* = 0.0066). Hydrogen treatment also enhanced the expression of glia marker GFAP in both cell lines (C6, 64.35 ± 5.69 vs. 22.87 ± 2.23, *p* = 0.0025; U87, 62.45 ± 3.20 vs. 12.51 ± 1.29, *p* = 0.0001) (Fig. 3a).

To further confirm the inhibitory effect of molecular hydrogen on the stemness of glioma cells, flow cytometry was used to determine the proportion of CD133-positive cells from both C6 and U87 cells. Compared to control, hydrogen treatment significantly decreased the proportion of CD133-positive cells in both C6 (15.39 ± 0.70 vs. 24.83 ± 1.30, *p* = 0.0031) and U87 (13.70 ± 0.97 vs. 33.98 ± 0.52, *p* < 0.0001) cells (Fig. 3b).

To investigate the effects of molecular hydrogen on cancer stem cell self-renewal capacity of glioma cells, we performed sphere formation assays. As shown in Fig. 3c and d, hydrogen treatment significantly inhibited the sphere-forming ability of the glioma cells, as evidenced by the decreased number (C6, 3.00 ± 1.15 vs. 11.33 ± 1.20, *p* = 0.0075; U87, 1.67 ± 0.33 vs. 15.00 ± 1.53, *p* = 0.001) and the smaller size of the tumor spheres.

### Hydrogen treatment promoted glioma stem-like cell (GSC) differentiation

**Fig. 4:**
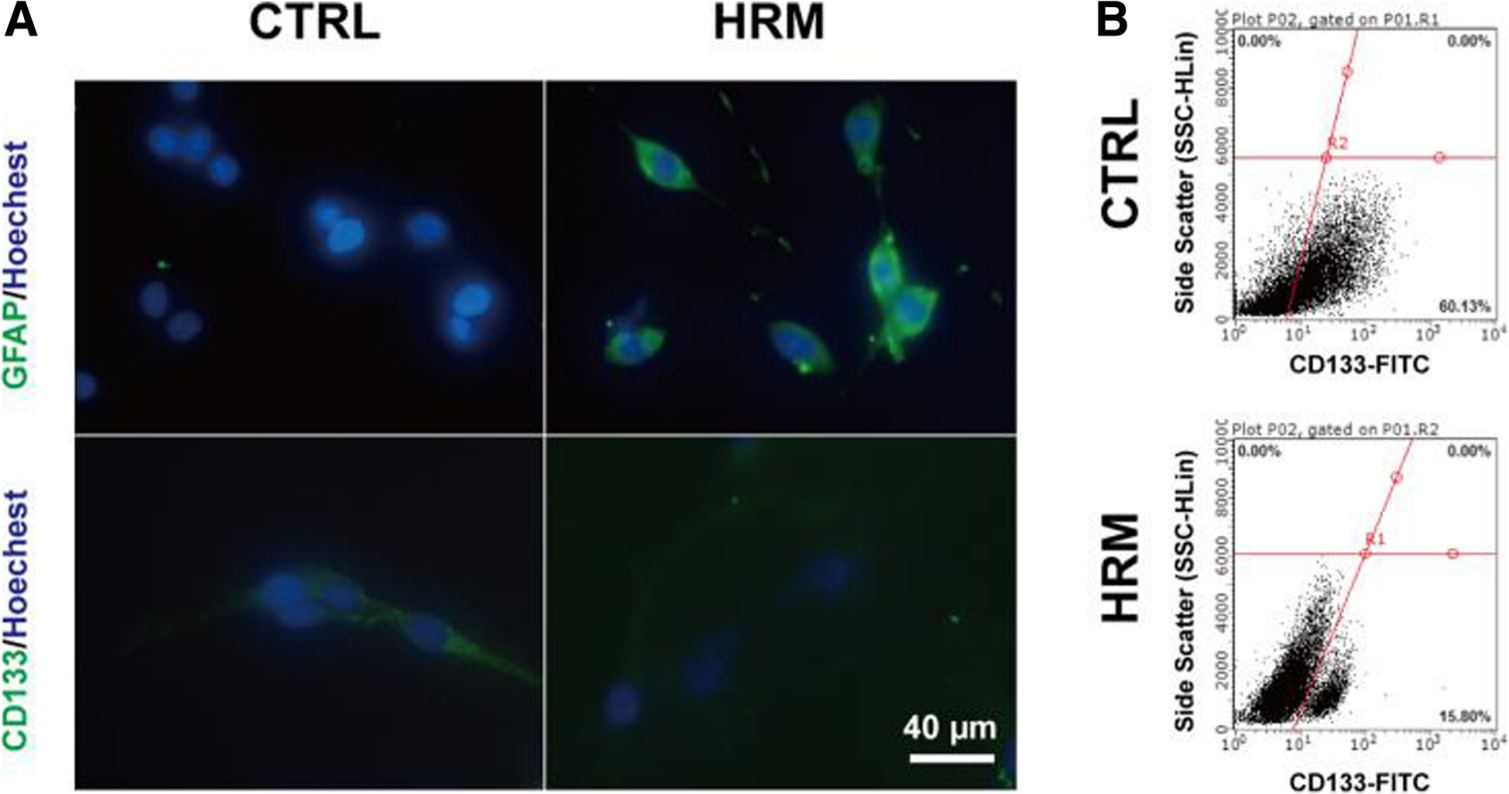
Hydrogen treatment induced the differentiation of C6 sub-spheres. The expression levels of GFAP and CD133 in C6 sub-spheres were assessed with immunofluorescence staining after hydrogen treatment (**a**)**.** The CD133-positive cell number was determined using flow cytometry analysis after hydrogen treatment (**b**). GFAP, glial fibrillary acidic protein; CTRL, control; HRM, hydrogen-rich medium

To directly determine the effects of hydrogen treatment on the GSCs, the sub-sphere formation assay and surface marker analysis were performed. C6 cells grew to form tumor spheres, and sub-spheres were formed 4–5 days after the primary sphere was dissociated. Immunofluorescence results showed that hydrogen treatment markedly increased the expression of GFAP (58.13 ± 6.63 vs. 18.60 ± 1.79, *p* = 0.0045) and significantly downregulated the expression of CD133 (13.43 ± 2.46 vs. 32.34 ± 6.14, *p* = 0.046) (Fig. 4a). Flow cytometry analysis showed that the CD133-positive cell number was significantly decreased in the hydrogen-treated group (Fig. 4b).

### Hydrogen treatment suppressed migration, invasion, and the colony-forming ability of glioma cells

**Fig. 5:**
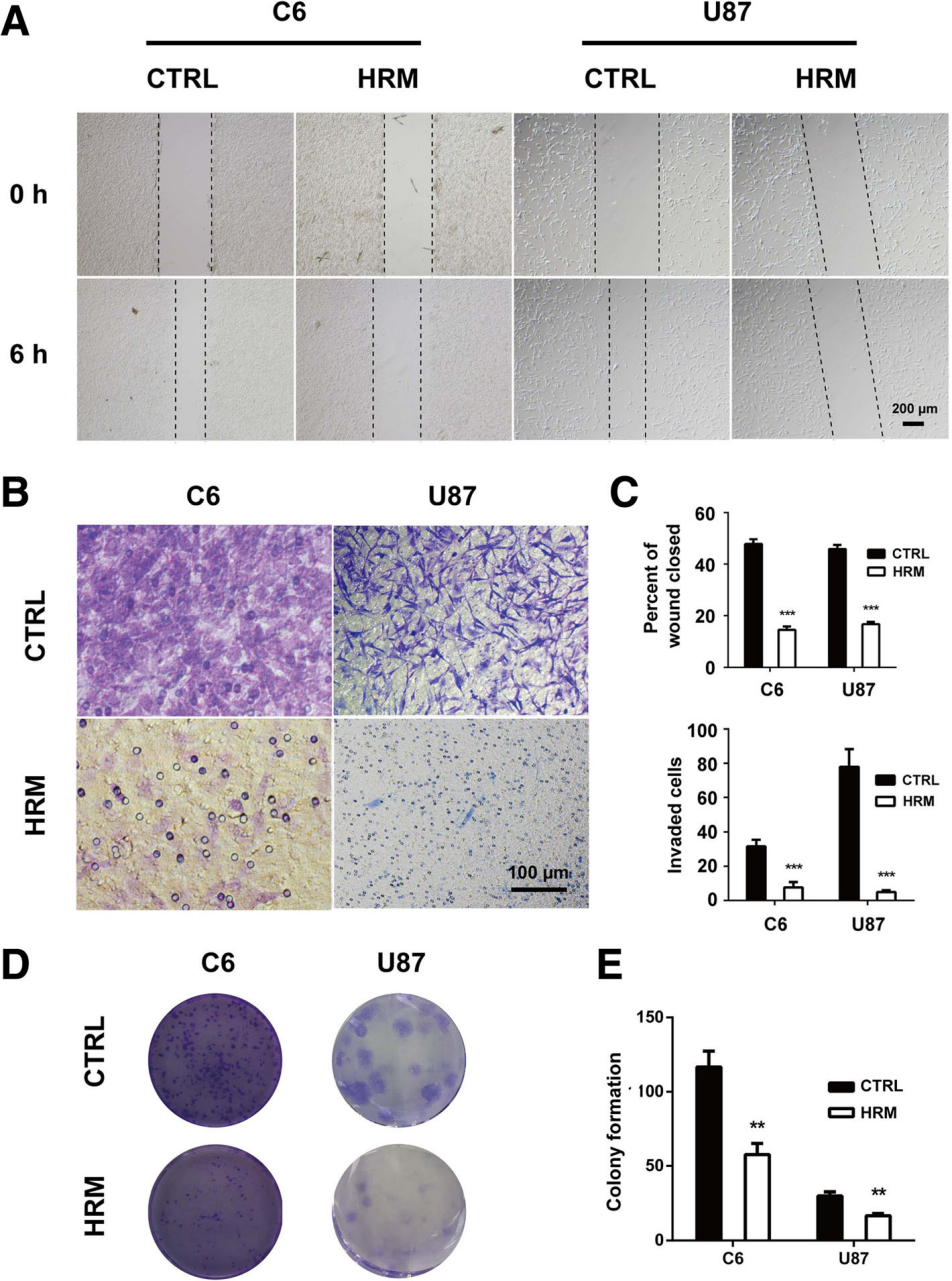
Hydrogen treatment inhibited the migration, invasion, and colony-forming ability of both C6 and U87 cells. The cell migration ability was examined using a wound-healing assay after treatment with hydrogen (**a**)**.** The cell invasion ability was determined using Matrigel invasion assay after treatment with hydrogen (**b**)**.** The quantification of the migration and invasion assay represents three independent experiments (**c**)**.** The colony-forming ability was evaluated using colony formation assay after hydrogen treatment (**d**). The quantification of the colony formation assay represents three independent experiments (**e**). **p < 0.01, ***p < 0.001 compared with the CTRL group. CTRL, control; HRM, hydrogen-rich medium

To identify the potential effects of molecular hydrogen on the migration of glioma cells, an in vitro wounding assay was employed. As shown in Fig. 5a and c, hydrogen treatment significantly decreased the motile nature of both C6 (14.47 ± 0.72 vs. 47.83 ± 1.05, *p* < 0.0001) and U87 cells (12.67 ± 0.50 vs. 33.68 ± 1.69, *p* < 0.0001).

In parallel, a matrigel invasion assay was also performed to determine the influence of hydrogen treatment on the invasion of glioma cells. As shown in Fig. 5b and c, molecular hydrogen markedly inhibited the cell invasiveness in both C6 (24.83 ± 1.30 vs. 33.98 ± 0.52, *p* < 0.0001) and U87 cells (24.83 ± 1.30 vs. 33.98 ± 0.52, *p* = 0.0005).

To determine the effect of hydrogen treatment on the colony-forming ability of glioma cells, the colony formation assay was performed. In both C6 and U87 cells, the number of colony-forming cells was markedly decreased in the hydrogen treatment group compared to the control (C6, 57.67 ± 4.37 vs. 116.70 ± 6.12, *p* = 0.0014; U87, 16.67 ± 0.88 vs. 30.00 ± 1.53, *p* = 0.0016) (Fig. 5d, e).

## Discussion

The effects of molecular hydrogen on cancer have been reported in several types of tumors including skin squamous cell carcinomas [7], lung cancer [9, 10], ovarian cancer [11], thymic lymphoma [12], liver tumors [13, 17], renal cell carcinoma [14], and colon cancer [16, 18]. However, whether molecular hydrogen has an anti-tumor effect on GBM has remained unknown. In the present study, we provide the first evidence that hydrogen treatment could exert an anti-tumor effect against GBM cells. Using the rat orthotopic glioma model and mouse subcutaneous xenograft model, we demonstrated that hydrogen inhalation could effectively suppress GBM tumor growth and prolong the survival of mice with GBM. This was further confirmed by IHC examination, which showed that hydrogen treatment suppressed the expression of the proliferation marker Ki67 and the angiogenesis marker CD34. IHC staining also showed that the expression of stemness markers was significantly decreased by hydrogen inhalation. This indicates that hydrogen treatment could attenuate the cancer stemness of glioma cells, which we further confirmed by the in vitro cell study. Hydrogen treatment suppresses the migration, invasion, and colony formation of glioma cells in vitro.

The grim prognosis for GBM is at least partly due to the lack of successful drug delivery across the blood–brain barrier (BBB) [19]. Due to its small molecular size and non-polarity, hydrogen gas can easily cross the BBB. This property along with its biological benefits makes hydrogen a promising candidate for the treatment of GBM. It should be noted that 67% of hydrogen gas and 33% oxygen gas was used in our in vivo study. The hydrogen and oxygen concentration was much higher than that used in most of the previous studies (≈ 1–4% H_2_ and 21% O_2_). Although the effects of low concentration of molecular hydrogen have been extensively reported in many kinds of disease, some studies have used high concentration of molecular hydrogen, which also have anti-tumor effects [7, 9, 11]. Taken together, these studies may suggest that a high concentration of molecular hydrogen may be more effective on the treatment of cancer; however, the dose-response relationship of molecular hydrogen needs to be further investigated.

Glioma stem-like cells (GSCs) are a rare subpopulation of GBM tumor cells with the ability of unlimited proliferation, self-renewal, and multipotent differentiation [20]. Those cells are responsible for glioma initiation, invasiveness, therapeutic resistance, and subsequent recurrence of tumors and are thus considered an essential target in GBM treatment [21, 22]. In this study, we demonstrated for the first time that hydrogen treatment could induce the differentiation of GSCs. Both in vivo and in vitro studies showed that hydrogen treatment could attenuate the stemness of glioma cells, which was evidenced by the decreased expression of stemness markers (CD133 and Nestin) and the enhanced expression of glia marker GFAP. The sphere-forming ability was also suppressed after hydrogen treatment. To directly assess the effects of hydrogen treatment on GSC differentiation, we examined the surface markers of the C6 subsphere cells. The results demonstrated that the expression of GFAP was elevated and CD133 expression was downregulated by hydrogen treatment. The flow cytometry analysis also showed a decreased number of CD133-positive cells in hydrogen-treated C6 subspheres.

A previous study showed that exposure of GSCs to serum could induce mitochondrial reactive oxygen species (ROS), as well as oxidative stress responses, leading to the appearance of differentiation morphology and downregulation of stemness markers [23]. ROS could also induce normal stem cell differentiation in some experimental systems [24]. Thus, it seems paradoxical that as an antioxidant, molecular hydrogen induces GSC differentiation. Previous studies have shown that molecular hydrogen can selectively scavenge the highly cytotoxic hydroxyl radicals; however, this explanation does not adequately explain the diverse effects of molecular hydrogen [25, 26]. For example, Kawasaki et al. reported that although molecular hydrogen did not reduce hydroxyl radicals in mesenchymal stem cells (MSCs), it did effectively prolong the in vitro replicative lifespan of bone marrow without losing differentiation potentials [27]. Intriguingly, molecular hydrogen has also been reported to act as a mitohormetic effector by mildly inducing mitochondrial superoxide production [28]. Perhaps hydrogen-induced ROS promoted the differentiation and downregulation of stemness in GSCs. However, the exact underlying mechanisms need to be further investigated.

We also examined the effect of molecular hydrogen on migration, invasion, and the colony-forming ability of GBM cells. The results showed that hydrogen treatment suppressed migration, invasion, and the colony-forming ability in both C6 and U87 cells. We reported similar effects in a previous study on ovarian cancer cells [11]. A hallmark of GBM is the extensive invasion of tumor cells into the normal brain tissues and along the vascular tracks, which prevents complete resection of all malignant cells [3, 29]. Frontline cytotoxic therapies, including chemotherapy and radiotherapy, are largely ineffective in halting GBM invasion [30]. Thus, the inhibitory effect of molecular hydrogen on the migration and invasion of glioma cells makes it a highly promising candidate as a potential therapeutic agent for the treatment of GBM, either alone or in combination with other anticancer therapeutics.

## Conclusion

The present study demonstrates that inhalation of 67% hydrogen gas has an inhibitory effect on GBM. Both in vivo and in vitro studies showed that molecular hydrogen could induce the desired differentiation of GSCs. Moreover, molecular hydrogen also suppresses the migration, invasion, and colony-forming ability of GBM cells. Therefore, our results suggest that molecular hydrogen may serve as a potential anti-tumor agent for treating GBM. Further research on the mechanisms and clinical investigations are highly warranted.

## Abbreviations

bFGF: Basic fibroblast growth factor
CTRL: Control group
EGF: Epidermal growth factor
GBM: Glioblastoma
GSCs: Glioma stem-like cells
HI: Hydrogen inhalation group
IHC: Immunohistochemistry
MSCs: Mesenchymal stem cells
NHE water: Neutral pH hydrogen-enriched electrolyzed water
ROS: Reactive oxygen species

## References

[1] Bastiancich C, Bastiat G, Lagarce F (2018). Gemcitabine and glioblastoma: challenges and current perspectives. Drug Discov Today.

[2] Reitman ZJ, Winkler F, Elia A (2018). New directions in the treatment of glioblastoma. Semin Neurol.

[3] Basso J, Miranda A, Sousa J, Pais A, Vitorino C (2018). Repurposing drugs for glioblastoma: from bench to bedside. Cancer Lett.

[4] Barnholtz-Sloan JS, Ostrom QT, Cote D (2018). Epidemiology of brain tumors. Neurol Clin.

[5] Ohsawa I, Ishikawa M, Takahashi K (2007). Hydrogen acts as a therapeutic antioxidant by selectively reducing cytotoxic oxygen radicals. Nat Med.

[6] Ichihara M, Sobue S, Ito M, Hirayama M, Ohno K (2015). Beneficial biological effects and the underlying mechanisms of molecular hydrogen – comprehensive review of 321 original articles. Med Gas Res..

[7] Dole M, Wilson F, Fife W (1975). Hyperbaric hydrogen therapy: a possible treatment for cancer. Science..

[8] Saitoh Y, Okayasu H, Xiao L, Harata Y, Miwa N (2008). Neutral pH hydrogen-enriched electrolyzed water achieves tumor-preferential clonal growth inhibition over normal cells and tumor invasion inhibition concurrently with intracellular oxidant repression. Oncol Res.

[9] Wang D, Wang L, Zhang Y, Zhao Y, Chen G (2018). Hydrogen gas inhibits lung cancer progression through targeting SMC3. Biomed Pharmacother.

[10] Jiang Y, Liu G, Zhang L (2018). Therapeutic efficacy of hydrogen-rich saline alone and in combination with PI3K inhibitor in non-small cell lung cancer. Mol Med Rep.

[11] Shang L, Xie F, Li J (2018). Therapeutic potential of molecular hydrogen in ovarian cancer. Transl Cancer Res.

[12] Zhao L, Zhou C, Zhang J (2011). Hydrogen protects mice from radiation induced thymic lymphoma in BALB/c mice. Int J Biol Sci.

[13] Kawai D, Takaki A, Nakatsuka A (2012). Hydrogen-rich water prevents progression of nonalcoholic steatohepatitis and accompanying hepatocarcinogenesis in mice. Hepatology..

[14] Li F, Zhu S, Wang Z, Wang H, Zhao Y, Chen G (2013). Consumption of hydrogen-rich water protects against ferric nitrilotriacetate-induced nephrotoxicity and early tumor promotional events in rats. Food Chem Toxicol.

[15] Nakashima-Kamimura N, Mori T, Ohsawa I, Asoh S, Ohta S (2009). Molecular hydrogen alleviates nephrotoxicity induced by an anti-cancer drug cisplatin without compromising anti-tumor activity in mice. Cancer Chemother Pharmacol.

[16] Yang Q, Ji G, Pan R, Zhao Y, Yan P (2017). Protective effect of hydrogen-rich water on liver function of colorectal cancer patients treated with mFOLFOX6 chemotherapy. Mol Clin Oncol.

[17] Kang K, Kang Y, Choi I (2011). Effects of drinking hydrogen-rich water on the quality of life of patients treated with radiotherapy for liver tumors. Med Gas Res.

[18] Runtuwene J, Amitani H, Amitani M, Asakawa A, Cheng K, Inui A (2015). Hydrogen-water enhances 5-fluorouracil-induced inhibition of colon cancer. Peer J.

[19] van Tellingen O, Yetkin-Arik B, Gooijer d, Wesseling P, Wurdinger T, de Vries H (2015). Overcoming the blood-brain tumor barrier for effective glioblastoma treatment. Drug Resist Update.

[20] Lathia J, Mack S, Mulkearns-Hubert E, Valentim C, Rich J (2015). Cancer stem cells in glioblastoma. Genes Dev.

[21] Sorensen M, Fosmark S, Hellwege S, Beier D, Kristensen B, Beier C (2015). Chemoresistance and chemotherapy targeting stem-like cells in malignant glioma. Adv Exp Med Biol.

[22] Cheng L, Bao S, Rich J (2010). Potential therapeutic implications of cancer stem cells in glioblastoma. Biochem Pharmacol.

[23] Yuan S, Lu Y, Yang J (2015). Metabolic activation of mitochondria in glioma stem cells promotes cancer development through a reactive oxygen species-mediated mechanism. Stem Cell Res Ther.

[24] Bigarella C, Liang R, Ghaffari S (2014). Stem cells and the impact of ROS signaling. Development.

[25] Wood K, Gladwin M (2007). The hydrogen highway to reperfusion therapy. Nat Med.

[26] Penders J, Kissner R, Koppenol W (2014). ONOOH does not react with H_2_: potential beneficial effects of H_2_, as an antioxidant by selective reaction with hydroxyl radicals and peroxynitrite. Free Radical Bio Med.

[27] Kawasaki H, Guan J, Tamama K (2010). Hydrogen gas treatment prolongs replicative lifespan of bone marrow multipotential stromal cells in vitro while preserving differentiation and paracrine potentials. Biochem Bioph Res Co.

[28] Murakami Y, Ito M, Ohsawa I (2017). Molecular hydrogen protects against oxidative stress-induced SH-SY5Y neuroblastoma cell death through the process of mitohormesis. PLoS One.

[29] Liu C, Chang C, Hsueh K (2018). Migration/invasion of malignant gliomas and implications for therapeutic treatment. Int J Mol Sci.

[30] Pencheva N, de Gooijer M, Vis D (2017). Identification of a druggable pathway controlling glioblastoma invasiveness. Cell Rep.

